# A pilot study of a mobile application for postural analysis and training support in Shotokan Karate

**DOI:** 10.1038/s41598-026-41414-5

**Published:** 2026-02-26

**Authors:** Carlos Manuel Silva, António Oseas Pataca, Frederico Branco, Paulo Jorge Coelho, Ivan Miguel Pires

**Affiliations:** 1 Escola Superior de Tecnologia e Gestão de Águeda, Universidade de Aveirot, Águeda, Portugal; 2 Instituto de Telecomunicações, Escola Superior de Tecnologia e Gestão de Águeda, Universidade de Aveiro, Águeda, Portugal; 3 Institute for Systems and Computer Engineering, Technology and Science (INESC TEC), 4200-465 Porto, Portugal; 4 School of Science and Technology, Universidade de Trás-os-Montes e Alto Douro, 5000-801 Vila Real, Portugal; 5 School of Technology and Management, Polytechnic University of Leiria, Leiria, Portugal; 6 Institute for Systems Engineering and Computers at Coimbra, University of Coimbra, Coimbra, Portugal

**Keywords:** Shotokan Karate, Image processing, Mobile devices, Postural analysis, Engineering, Health care, Mathematics and computing

## Abstract

This paper presents a smartphone application that supports Shotokan Karate training by analysing posture and providing real-time feedback. The app evaluates three fundamental stances (Zenkutsu Dachi, Kokutsu Dachi, and Kiba Dachi) using Google ML Kit Pose Detection to extract body landmarks and compute joint-angle and alignment features, including proxy indicators of weight shift. The app also includes conditioning exercises (squats and push-ups) and a reflex-oriented interaction task. Results from a single-participant pilot are reported as feasibility evidence only and should not be generalised. A larger validation study with at least 30 practitioners across three skill levels (beginner, intermediate, advanced) is required, together with power analysis and reliability assessment, before broader conclusions can be drawn.

## Introduction

Karate is practised worldwide and emphasises posture, body control, and stance execution as foundations for performance and safety^1,2^. In Shotokan Karate, stance quality is central to effective technique and is commonly assessed through instructor observation and correction^1,3,4^.

Technique assessment in karate is primarily instructor-driven and therefore subjective and context-dependent^1,5^. This creates a practical limitation for unsupervised training, where practitioners have limited access to consistent, objective feedback on subtle postural deviations^4,6,7^.

Mobile computing and markerless pose estimation enable low-cost movement analysis outside laboratory settings and have been adopted in rehabilitation and fitness^8,9^. Comparable approaches remain less explored in martial arts training, particularly for stance-specific feedback using consumer smartphones^4,6,10–12^.

This paper presents a smartphone application that uses Google ML Kit Pose Detection to extract body landmarks and compute joint-angle and alignment features from a lateral camera view^13^. A rule-based decision layer evaluates three fundamental Shotokan stances: Zenkutsu Dachi, Kokutsu Dachi, and Kiba Dachi^1,3,4,6,7^. The application also includes a small set of conditioning tasks (squats and push-ups) and a reflex-oriented interaction task that reuses the same pose landmarks for repetition detection and event timing^12,13^.

The goal is to augment, not replace, instructor feedback by providing stance-oriented cues during independent practice^4,5,8^. The intended contribution is feasibility evidence that stance criteria can be operationalised with on-device pose estimation under controlled capture^4,8,9,11,12^.

The remainder of the paper describes the system design, the stance evaluation rules, and a pilot feasibility evaluation. We report descriptive results, discuss limitations of single-view pose estimation and rule-based thresholds, and outline a structured multi-participant validation protocol.

While this study offers promising insights into the application of smartphone-based technology for Shotokan Karate training, it is important to note that the quantitative evaluation is based on a single participant. As such, the results should be regarded as preliminary and illustrative, rather than conclusive findings. The purpose of this feasibility pilot study is to explore the feasibility of the approach, and further validation with a larger sample size with a minimum of 30 Shotokan Karate practitioners across three skill strata (beginner, intermediate, advanced) is required before making broader claims about the app’s efficacy. Future studies will also incorporate statistical power analysis and reliability assessments to ensure more reliable and generalisable results.

## Related work

Markerless motion capture (MMC) has rapidly evolved from laboratory prototypes into practical tools for biomechanics, clinical movement assessment, and sports analysis, largely due to advances in deep learning based pose estimation and improved multi-view reconstruction pipelines. Unlike marker-based optical motion capture, MMC reduces preparation time and equipment cost, enabling movement capture in more natural environments and at larger scales. Recent survey work emphasises that MMC can provide clinically and biomechanically relevant kinematic estimates, while also noting persistent limitations such as sensitivity to occlusion, camera placement, and reduced accuracy in frontal and transverse plane rotations compared to sagittal plane measures^14,15^.

Several journal studies have validated modern MMC systems against marker-based gold standards for common functional tasks. For example, OpenCap estimates 3D kinematics and kinetics from videos recorded on consumer mobile devices and was validated against laboratory motion capture and force plates, reporting mean absolute errors of a few degrees for joint angles in controlled tests^16^. Independent validation studies further examined OpenCap for return-to-sport movements, again comparing against optoelectronic marker-based systems and reporting task-dependent agreement across hip, knee, and ankle kinematics^17^. In parallel, studies evaluating commercial markerless platforms such as Theia3D compared markerless and marker-based outputs during postural control tasks, supporting the broader feasibility of MMC beyond gait and highlighting where discrepancies remain^18^. Additional comparative work has proposed statistical agreement frameworks, such as functional limits of agreement, to evaluate waveform similarity between markerless and marker-based kinematics during exercises like lunges^19^.

Community datasets and clinical translation efforts are also strengthening the field. For instance, the BioCV dataset synchronises high-speed multi-camera video with optical marker trajectories and force plate measurements to facilitate rigorous development and benchmarking of markerless human movement analysis methods^20^. Finally, clinical and rehabilitation-focused reviews show accelerating adoption of MMC for measurement in patient populations, while stressing the need for standardised validation protocols, reporting conventions, and task-specific performance characterisation^21,22^.

In martial arts, technology-assisted assessment has primarily relied on wearables, inertial sensors, or laboratory instrumentation. Mobile, camera-based solutions remain less common, and many systems focus on instructional guidance rather than stance-specific postural measurement.

Recent developments in sensor-based technologies and artificial intelligence have opened opportunities for more quantitative and accessible analysis of physical performance. A systematic review of literature from 2015 to 2025 highlights a growing interest in using technological tools for movement evaluation in martial arts, particularly in Shotokan Karate, which emphasises posture, stance, and execution precision.

Several studies have demonstrated the use of kinematic sensors to evaluate motion in combat sports. Vuković et al.^23^ investigated the accuracy of sensor-based analysis of the gyaku tsuki (reverse punch), providing valuable data on execution variability and device reliability. Similarly, studies in Taekwondo^24^ and Wushu^25^ employed accelerometers, gyroscopes, and optical tracking systems to objectively assess performance, simulate movements, and enhance teaching through 3D visualisation.

In robotics, Li et al.^26^ explored the replication of Tai Chi movements using data captured by Kinect sensors, suggesting that technology can help preserve traditional movement systems. Jaysrichai et al.^27^ developed a reaction-time measurement system for strikes using sensors and visual feedback, while Venkatraman et al.^28^ focused on head-impact analysis through instrumented mouthguards, demonstrating the broader applications of sensor-based monitoring in combat sports.

A key gap is the lack of stance-focused, smartphone-only systems that provide interpretable, stance-specific feedback aligned with karate teaching criteria. Motivated by this, we developed a mobile application that uses only a smartphone camera and on-device machine learning (Google ML Kit Pose Detection) to evaluate Zenkutsu Dachi, Kokutsu Dachi, and Kiba Dachi in real time^13^. This approach enables real-time, self-guided correction of technique, introducing a level of automation and objectivity to traditional karate practice that has not been widely implemented in previous work.

Overall, prior work supports the broader feasibility of low-cost movement analysis but provides limited evidence for mobile, stance-oriented karate training tools. This study contributes with a feasibility pilot study implementation and an initial feasibility evaluation for three Shotokan stances.

## System architecture and design

The application comprises four functional modules: stance analysis, conditioning exercise tracking, a reflex-oriented interaction task, and session statistics. All processing is performed on the device using camera input and pose landmarks.

### Technological stack

The application was built using Flutter^29^, an open-source UI software development kit from Google. Flutter enables cross-platform development from a single codebase, allowing deployment on both Android and iOS devices. The programming logic was implemented in Dart, which integrates seamlessly with Flutter’s widget-based architecture, enabling efficient rendering and responsive user interfaces. Flutter was chosen because it offers consistent graphical interfaces across platforms, integrates smoothly with external libraries and APIs, and is supported by a robust developer community with comprehensive documentation.

### Pose detection engine

**Fig. 1:**
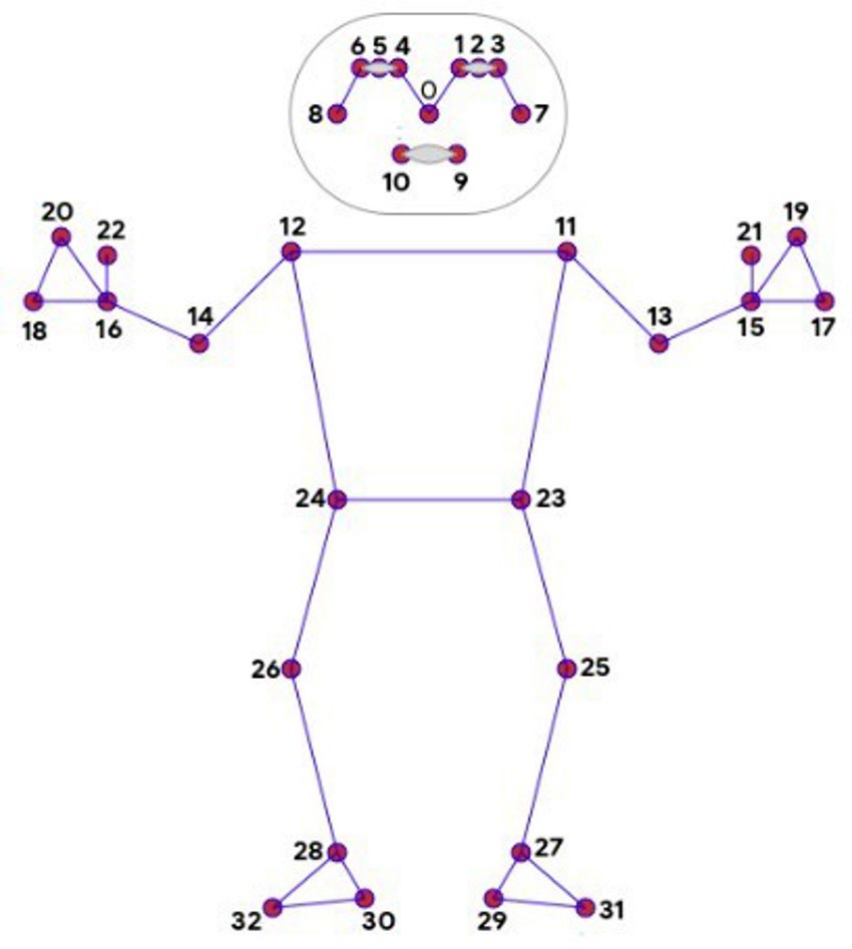
ML kit pose detection^13^.

At the core of the application’s technical functionality lies Google’s ML Kit—Pose Detection^13^. This machine learning API detects 33 anatomical key points from images or real-time video input. Each point is mapped with X, Y, and Z coordinates, enabling detailed estimation of body poses, as illustrated in Fig. 1.

Google ML Kit Pose Detection provides 33 full-body landmarks per frame (including facial and foot landmarks) and is designed for on-device, real-time inference. The ML Kit pose pipeline is based on Google’s BlazePose family of models, introduced initially for mobile, real-time fitness applications. BlazePose is optimised for speed on smartphones, but like other single-camera RGB pose estimators, it is sensitive to factors such as occlusion, motion blur, unusual viewpoints, and out-of-plane rotation. In practice, these conditions can lead to landmark jitter, intermittent keypoint swaps (e.g., left versus right in extreme poses), and increased angular error when joint angles are computed from noisy landmark coordinates.

Validation studies of BlazePose, the model family behind ML Kit Pose Detection, show that joint-angle errors can be low under good capture conditions^30^. However, these errors still depend on the type of movement and the camera angle. For example, Srinivasan et al. found an average mean absolute error of 2.11 degrees (RMSE 2.63 degrees) for hip, knee, and ankle angles during outdoor running when comparing BlazePose estimates to 2D Kinovea measurements^31^. Errors were slightly higher during faster movements, such as toe-off. These results should not be seen as a universal error limit for all movements or camera setups, since angular error can increase with occlusion, out-of-plane rotation, and motion blur.

ML Kit offers both a base (faster) and an accurate (slower) configuration, and it notes that the precision of landmark coordinates can vary^13^. This is another reason to report results as feasibility outcomes and to validate them against instrumented references in future studies.

To reduce instability in video mode, we used ML Kit’s streaming configuration, which tracks the most prominent person across frames and avoids running full person detection at every frame unless tracking confidence degrades. This tracking behaviour reduces latency and tends to improve the temporal stability of landmark trajectories compared to treating each frame independently.

Using these coordinates, the system calculates joint angles (hip, knee, and ankle), estimates weight distribution based on torso alignment, and evaluates postural alignment using geometric intersections. These measurements allow the app to determine whether a user is correctly performing fundamental Shotokan Karate stances such as Zenkutsu Dachi (forward stance), Kokutsu Dachi (backward stance), and Kiba Dachi (horse stance). Each stance has predefined criteria based on anatomical alignment, limb extension, and estimated weight shift, which are translated into algorithmic validation rules.

### Application structure and use cases

**Fig. 2:**
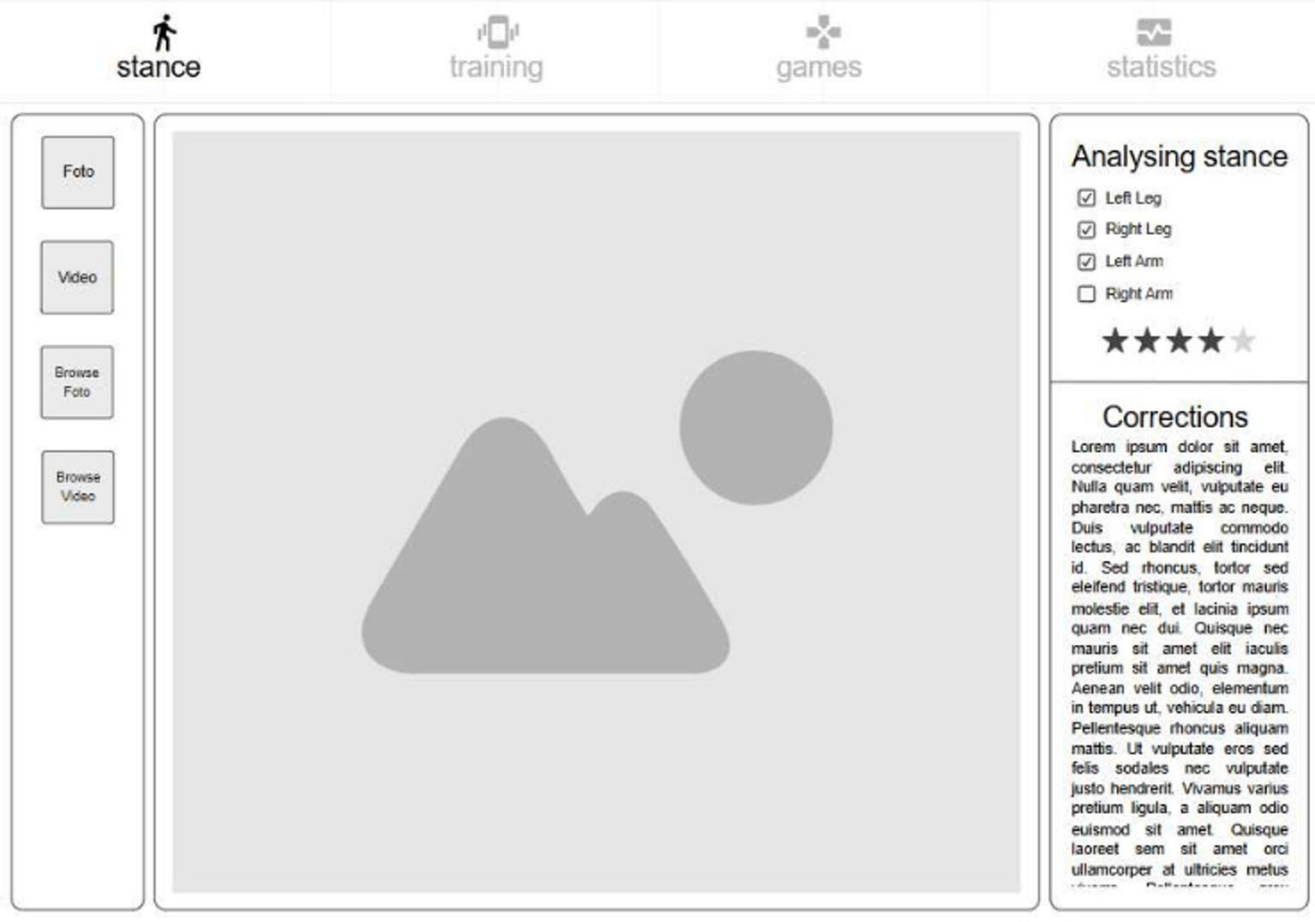
Posture analysis screen.

**Fig. 3:**
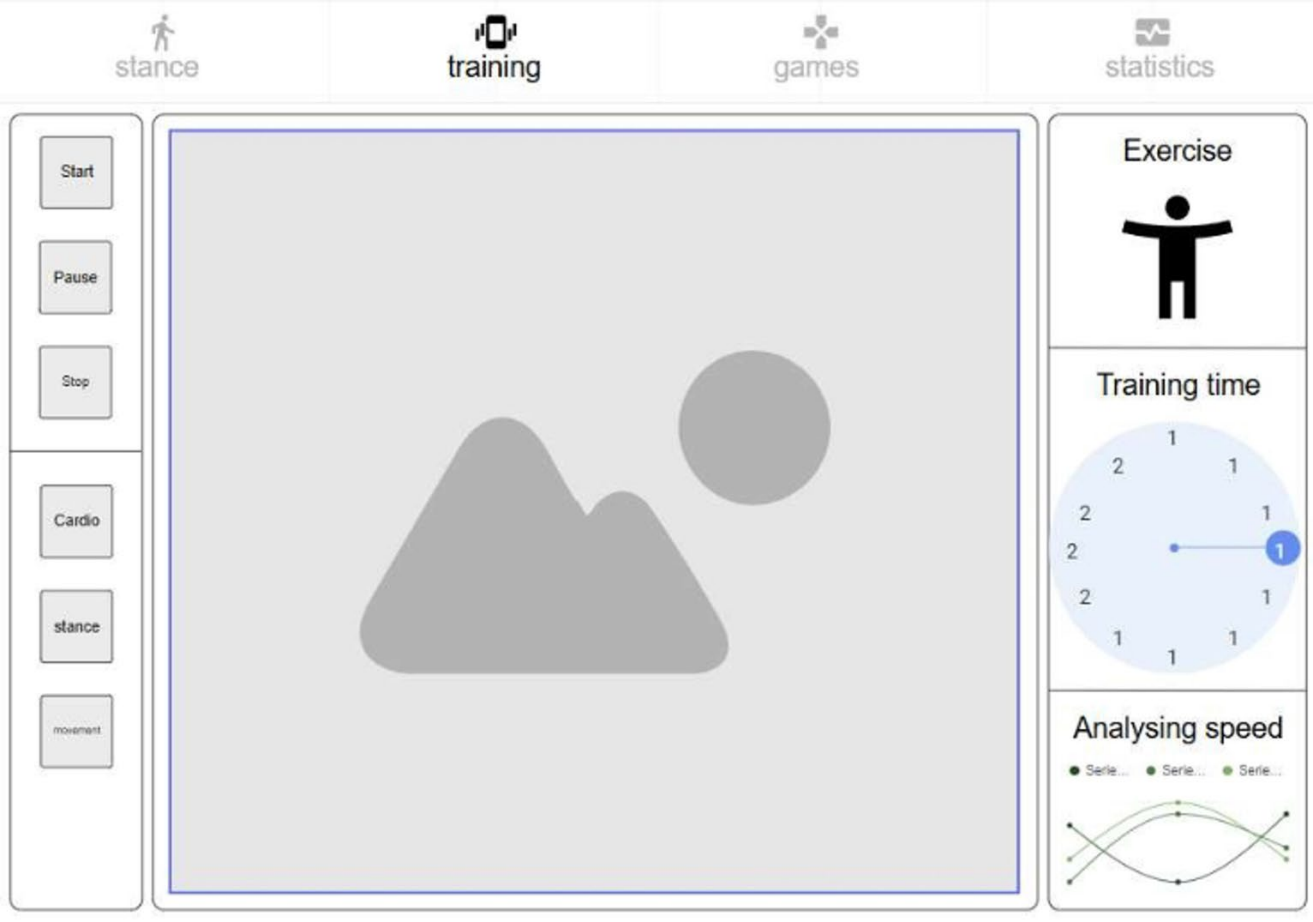
Training screen.

**Fig. 4:**
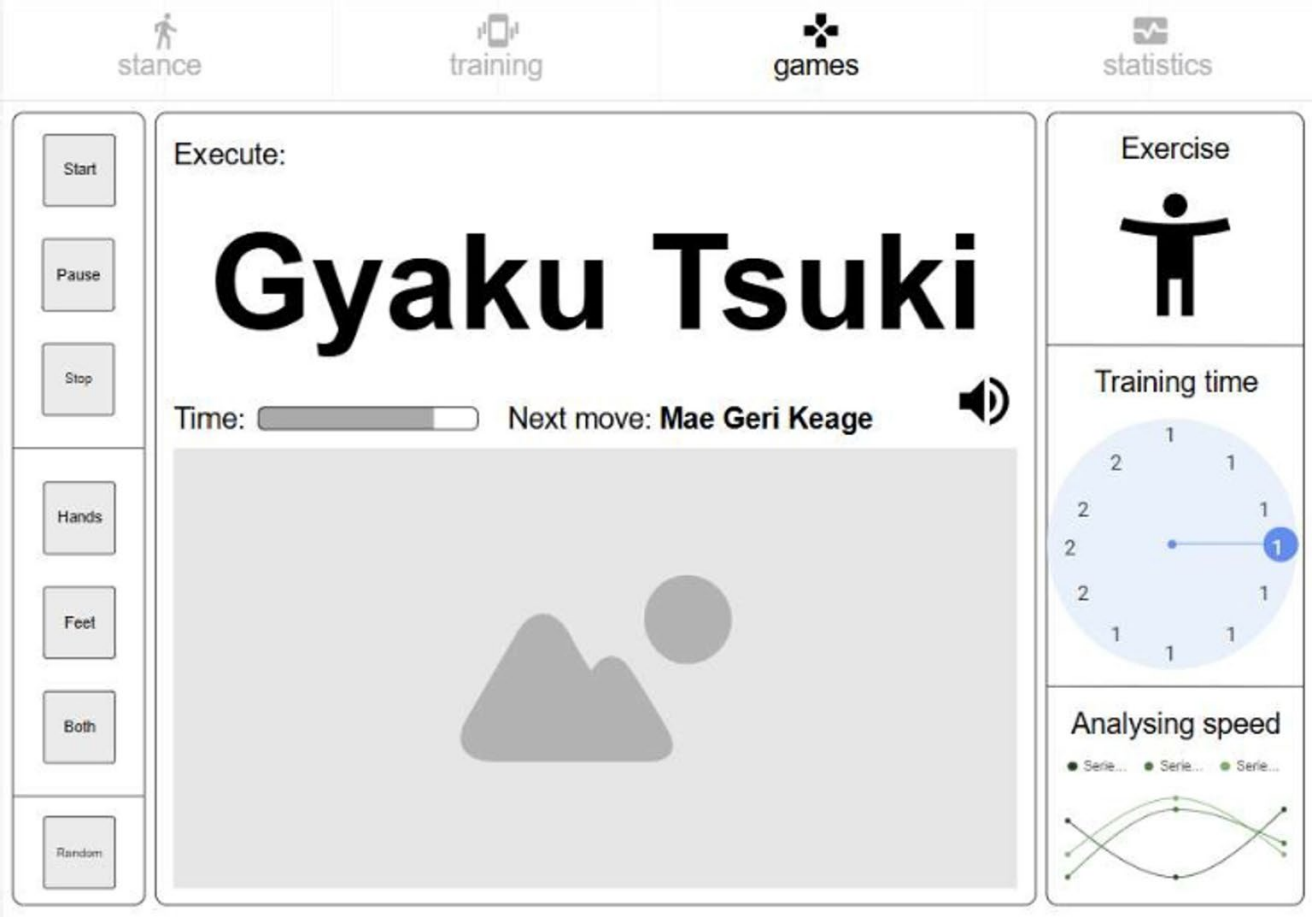
Games screen.

**Fig. 5:**
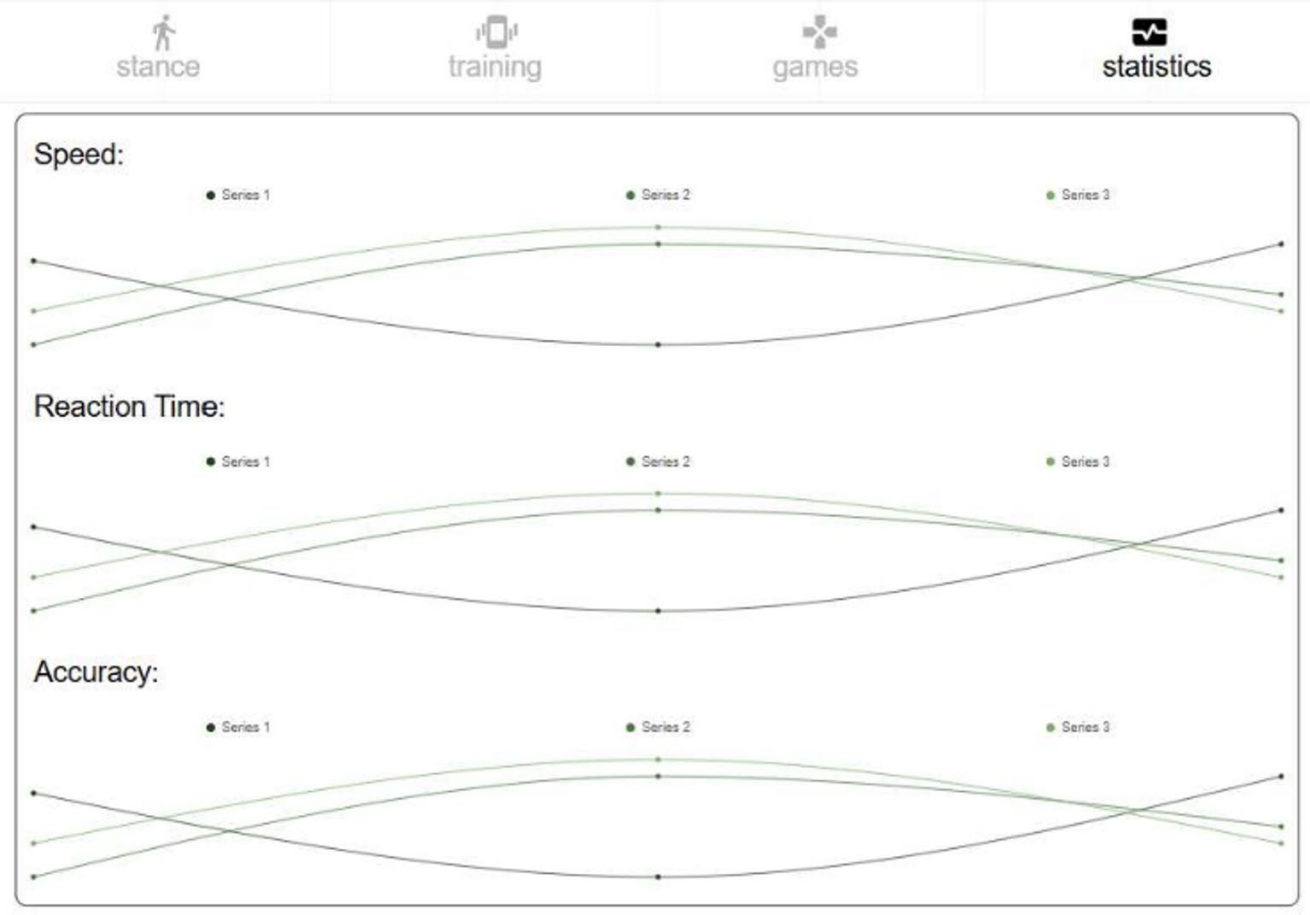
Statistics screen.

The app is structured around four primary modules, each described through use cases:

1. *Posture analysis module* Allows users to capture an image or use real-time video to analyse a karate stance. The application processes the image, detects key points, and provides feedback based on calculated angles and body alignment, as depicted in Fig. 2.
2. *Training module* Provides simple bodyweight exercises (e.g., push-ups and squats), with pose detection verifying movement correctness and counting repetitions based on limb angle transitions. Figure 3 presents the layout of the developed module.
3. *Games module* Implements a reflex test game where users are prompted to perform a punch toward a virtual target, as presented in Fig. 4. The application measures the reaction time; however, limitations in frame rate (30 fps) affect its responsiveness.
4. *Statistics module*: Stores historical data from training and game sessions. Figure 5 illustrates an example of the performance trend results, including max repetitions and cumulative progress.

### System workflow

**Fig. 6:**
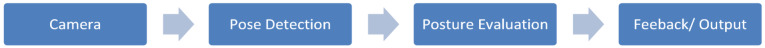
Workflow diagram.

The workflow, depicted in Fig. 6, consists of camera acquisition, pose landmark extraction, feature computation (joint angles and alignment proxies), and rule-based stance or exercise decisions. Feedback is rendered in real time, and session summaries are optionally stored for later review. The current pilot reports feasibility outcomes but does not include formal latency profiling.

### Data management, privacy, and user control

The application stores only session-level training summaries required to display progress statistics, specifically repetition counts, stance validation outcomes, and timestamps. No raw video is uploaded or retained by the system during the operation; pose keypoints and intermediate per-frame results are processed on the device and discarded after each session. Session summaries are stored locally on the user’s device and can be deleted at any time from within the application settings. Social sharing is optional and disabled by default; when enabled, the user explicitly selects the content to share and the target platform. The application does not post automatically or transmit training logs to third parties, except when the user initiates a share action through the operating system’s share interface. The app requests only the minimum device permissions required for camera capture and local storage of session summaries; no background collection is performed.

## Methodology

The methodology adopted in this project combines software engineering practices, computer vision, and biomechanical modelling to support karate training through a mobile application. The primary objective was to assess the correctness of karate stances using real-time image and video processing on standard mobile devices. This section outlines the methods used for posture analysis, training validation, and gameplay mechanics, as well as the underlying pose estimation techniques and decision rules implemented through algorithmic logic. The images presented for the training-exercise examples were performed by one of the authors, who provided consent for their publication.

The initial phase of development involved defining use cases to structure the application’s features. These use cases guided the functional implementation of four main features: posture analysis, training exercises (push-ups and squats), reflex-based games, and performance statistics and feedback.

The initial pilot test involved a single participant to assess the app’s feasibility and basic functionality. The tests were carried out in a controlled indoor environment, with standardised lighting conditions (300 lx), and the camera was positioned at 1.5 m from the participant at a 90-degree angle. The participant performed three core Karate stances (Zenkutsu Dachi, Kokutsu Dachi, and Kiba Dachi), each held for 10 s, followed by push-ups and squats for conditioning. For future studies, we propose increasing the sample size to more than 30 Shotokan Karate practitioners across three skill strata (beginner, intermediate, advanced) to ensure adequate statistical power. A power analysis will be conducted using G*Power or a similar software package to determine the appropriate sample size based on effect sizes from related studies in the literature. This will allow for more reliable and generalisable findings.

### Pose estimation and data collection

The key enabler of the application’s functionality is Google’s ML Kit Pose Detection API^13^, which identifies 33 key anatomical landmarks in both real-time video and still images. Each landmark is mapped with X and Y coordinates relative to the image origin in the top-left corner, and a Z coordinate that provides an experimentally calculated depth indication. This information enables the calculation of straight-line segments between joints (e.g., hip–knee–ankle) and supports angular analysis through vector operations.

In this study, joint angles were computed from ML Kit landmark coordinates using standard vector geometry. Because landmark localisation error propagates into angular estimates, the largest deviations are expected when joints are partially occluded, when limbs are aligned with the camera axis, or when the practitioner rotates outside the imaging plane. Additionally, the Z coordinate provided by ML Kit should be interpreted as a relative depth indication rather than a metric 3D reconstruction; therefore, the present work focuses on 2D angle features derived from the lateral view.

To mitigate pose-estimation noise, we applied basic quality control during analysis by requiring sufficient landmark confidence for the joints involved in each angle calculation, and by treating frames with low-confidence keypoints as unreliable for quantitative comparison. In future validation studies, we will incorporate explicit temporal filtering of landmark trajectories (e.g., moving-average, median, or one-euro-style smoothing) and report how filtering affects angular error and stance classification agreement. This aligns with the intended use of BlazePose as a real-time tracker but also acknowledges that biomechanics-focused accuracy evaluation requires explicit treatment of jitter and uncertainty.

For testing, the application was deployed and evaluated on both Android and iOS platforms. Specifically, the devices used were a Samsung Galaxy S10 (with Snapdragon 855, 6GB RAM) for Android, and an iPhone 12 (with A14 Bionic chip, 4GB RAM) for iOS. The tests were conducted in a controlled indoor environment with minimal background clutter to ensure optimal camera performance as previously described. The participant was an adult volunteer with no reported injuries or medical conditions, and no personal identifying information beyond video imagery was collected. To replicate this setup, future studies or practitioners should adhere to the specific distance, angle, and lighting level for consistent results.

### Planned multi-participant validation protocol

The present study is intended as a feasibility pilot study evaluation under controlled conditions. To assess external validity and generalizability, a follow-up validation study is planned with multiple participants and systematically varied capture conditions.

We plan to recruit more than 30 Shotokan Karate practitioners across three skill strata (beginner, intermediate, advanced) to capture variability in technique. Anthropometric diversity will be recorded using height, mass, and lower-limb segment lengths, and recruitment will aim to cover a broad range representative of the local practitioner population. Inclusion criteria will include age 18 years or older, regular Shotokan practice, and the ability to perform the three target stances safely. Exclusion criteria will include acute musculoskeletal injury, pain during stance execution, or any condition that limits safe participation.

Data will be collected in both controlled and realistic settings. Each participant will perform Zenkutsu Dachi, Kokutsu Dachi, and Kiba Dachi in (i) a controlled indoor setup and (ii) a dojo-like environment. Camera placement will include a standardised lateral view at a 1.5 m distance and 90º orientation, and additional variations will be recorded to quantify sensitivity to real-world capture differences (e.g., slight deviations in camera angle, distance, and lighting). Each stance will be held for a fixed duration and repeated across multiple trials. Device model, frame rate, resolution, and lighting conditions will be logged for each recording.

In future validation studies, stance labels and joint-angle measurements will be obtained from at least two independent Shotokan Karate instructors using a standardised annotation protocol that defines anatomical landmarks and angle conventions. App-estimated knee and shin angles will be compared to expert measurements using agreement and error metrics, including RMSE, Bland–Altman analysis, correlation, and confidence intervals.

Results will be reported subject-wise, summarising angle error and stance agreement per participant, then aggregating across participants. Confidence intervals will be reported for key summary metrics. Frames will not be treated as independent samples for inferential claims, and per-frame results will be used only as repeated measures within each participant.

Future work will include a structured usability study using a standard instrument (for example, SUS or UEQ) with a defined task set and participant count. It will report summary scores with confidence intervals.

### Evaluation protocol and reproducibility

This study includes two distinct evaluation methods. First, iterative engineering tests were performed during development to verify correct app behaviour and user interface feedback. Second, the quantitative values reported in the Results were computed from a predefined set of frames extracted from pilot recordings and compared against expert reference annotations.

For the quantitative comparison, frames were sampled from the recorded stance trials and annotated offline. Each annotated frame constitutes a single experimental unit for angle error calculations. Knee and shin angles were computed from ML Kit landmark coordinates using vector geometry, and errors were computed as the absolute differences between app-derived angles and expert reference angles. Frames with insufficient landmark confidence for the involved joints were excluded from the quantitative comparison.

To support reproducibility, all evaluation calculations were performed using a scripted analysis workflow that takes as input (i) the extracted frame set, (ii) the corresponding app output angles, and (iii) the expert reference annotations, and produces summary statistics. Because this is a feasibility-oriented pilot with a single participant and a single annotator, this workflow and the reported values are descriptive and illustrative rather than validated performance measures. A future multi-participant validation will use blinded, independent annotators, repeated annotations for reliability estimation, and agreement metrics such as RMSE and Bland-Altman analysis.

### Posture analysis algorithms

The evaluation of karate stances was achieved by formalising traditional teaching criteria into computational rules. For each stance (Zenkutsu Dachi, Kokutsu Dachi, Kiba Dachi), a specific algorithm was created to analyse:

1. *Weight distribution* Estimated by calculating the intersection point of the torso’s vertical axis (from shoulders to hips) with the foot support base.
2. *Limb angles* Computed between joints to assess leg flexion, hip extension, and arm position.
3. *Vision-based criteria* Such as the “invisible big toe” rule used in Zenkutsu Dachi, evaluated through a projected line of sight from the eyes to the toe.

The weight distribution and invisible big toe criteria are implemented as heuristic proxies derived from 2D keypoints rather than direct biomechanical measurements. Weight distribution is approximated by the relative position of the trunk’s vertical axis with respect to the base of support. It should be interpreted as a qualitative indicator of forward versus backward bias, not as a measure of ground reaction forces or true plantar loading. Similarly, the invisible big toe criterion is operationalised as a lateral-view geometric alignment check that is sensitive to camera perspective, out-of-plane rotation, and occlusion. In this pilot study, these two criteria were not independently validated against force measurements or a dedicated expert-scored ground truth. Future work will validate each proxy using expert labels and agreement metrics and will quantify sensitivity to camera placement and perspective deviations.

**Fig. 7:**
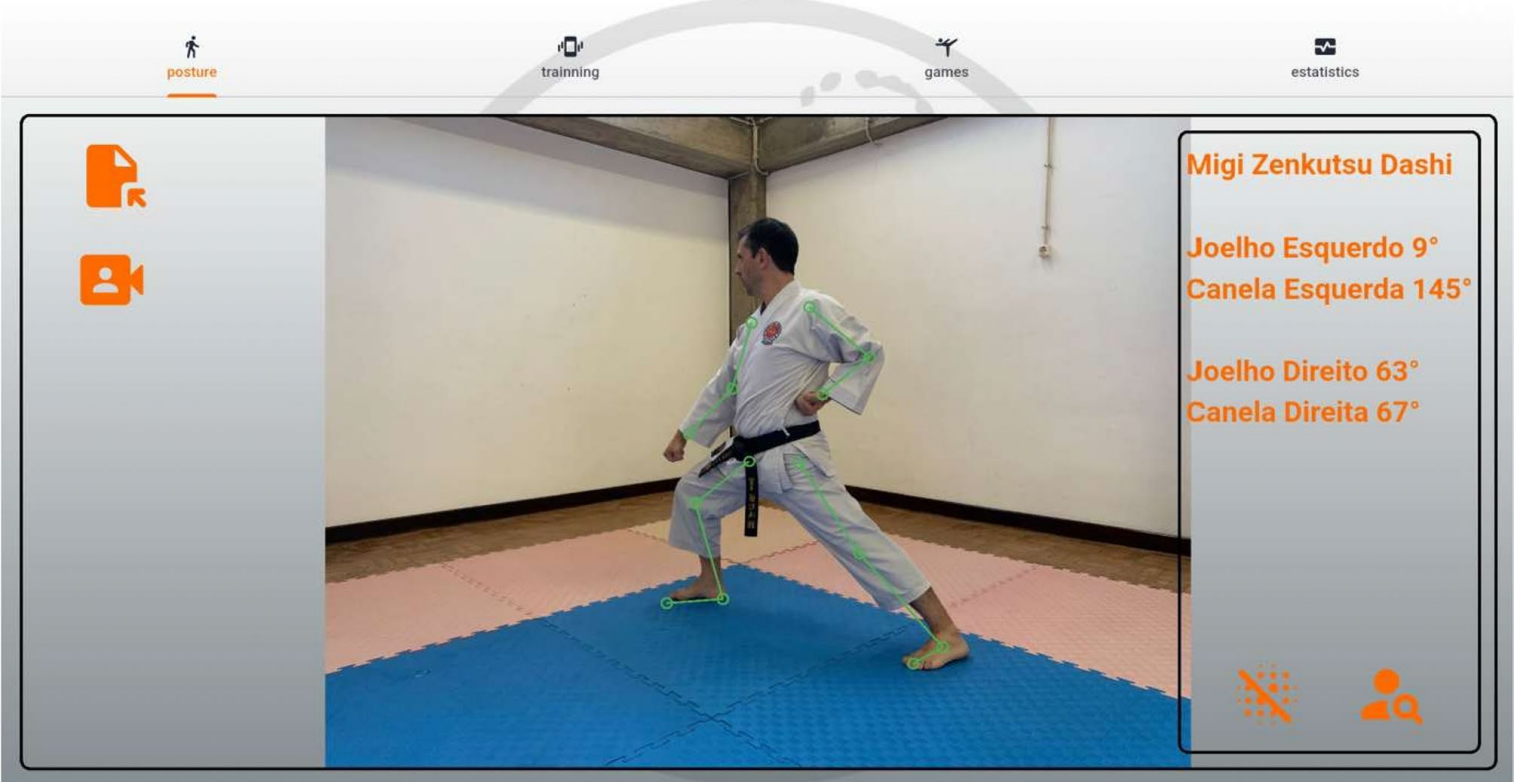
Pose detection and analysis.

As presented in Fig. 7, the Zenkutsu Dachi detection algorithm identifies:

- The front leg is based on the horizontal foot distance.
- Trunk alignment based on the shoulder–hip axis.
- Whether the knee bends past the toes by tracing a projected visual line.

All position-checking logic is mathematically grounded using geometric operations (e.g., line intersection formulas, scalar product for angle calculation).

The angular thresholds for posture correctness were determined using a combination of empirical tuning, expert input, and literature-derived guidelines. The initial empirical tuning was performed during development using exploratory tests and expert feedback. Nevertheless, the formal evaluation reported in this study involved a single participant. The joint angles and body alignments were reviewed against expert feedback from a Shotokan Karate instructor to assess alignment with traditional stance criteria. Expert stance criteria and iterative exploratory tuning over development informed the initial threshold ranges. The appropriate literature was used to guide the plausibility of the kinematic features and the general feasibility of sensor-based or vision-based measurement in martial arts contexts, rather than to define definitive stance-specific numeric cutoffs^23^. These thresholds were fine-tuned through exploratory testing under controlled conditions during development.

### Threshold definitions and stance decision rules

Stance classification was implemented as a rule-based procedure that translates traditional Shotokan stance criteria into a set of measurable features computed from ML Kit keypoints. The app evaluates joint angles and alignment relationships (e.g., relative knee flexion between legs, shin orientation, trunk lean relative to the vertical axis, and stance width based on foot separation). It applies a small number of threshold conditions to assign one of the three stance labels. The angular thresholds used in this study are stance-specific and context-dependent, representing a computational formalisation of Shotokan Karate teaching criteria rather than universal biomechanical norms.

In this feasibility pilot study implementation, thresholds were initialised from expert-informed stance criteria and then refined through exploratory tuning under controlled conditions (fixed lateral camera placement, approximately perpendicular body orientation, stable lighting, and static background). Because the formal quantitative evaluation reported in this study involved a single participant, these thresholds should be interpreted as preliminary implementation parameters rather than validated biomechanical cutoffs. Below, we express each rule as a conjunction of threshold predicates, and we define each variable used in the formulas.

For reproducibility, the rule structure used by the classifier is summarised below:

- *Zenkutsu Dachi* Classified when the front leg exhibits greater flexion than the rear leg, together with an upright trunk constraint within a tolerance window, and minimum stance separation to reduce ambiguity. It is represented as:
  $$\:ZD\:\iff\:\:(F\_front\:>\:F\_back)\:AND\:(theta\_body\:in\:T\_upright)\:AND\:(D\_feet\:\ge\:\:D\_min)$$
  where:
  - F_front, F_back: knee flexion (hip-knee-ankle angle) for front and rear legs.
  - theta_body: trunk inclination.
  - T_upright: allowed trunk-tilt tolerance window.
  - D_feet: foot separation.
  - D_min: minimum separation threshold.
- *Kokutsu Dachi* Classified when the rear leg exhibits greater flexion than the front leg, together with an upright trunk constraint within a tolerance window, and a posterior weight shift proxy derived from torso alignment relative to the base of support. It is represented as:
  $$\:KD\:\iff\:\:(F\_back\:>\:F\_front)\:AND\:(theta\_body\:in\:T\_upright)\:AND\:(W\_back\:\ge\:\:W\_min)$$
  where:
  - W_back is the posterior weight-shift proxy derived from the torso vertical axis relative to the base of support (as you already describe conceptually earlier).
  - W_min is the minimum posterior shift required to classify as Kokutsu Dachi.
  - Other terms as defined above.
- *Kiba Dachi* Classified when both knees exhibit flexion within a target range, and the stance width exceeds a minimum separation threshold, reflecting a laterally symmetric base of support.
  $$\:KiD\:\iff\:\:(F\_left\:in\:F\_range)\:AND\:(F\_right\:in\:F\_range)\:AND\:(D\_feet\:\ge\:\:D\_kiba\_min)$$
  where:
  - F_left, F_right are the left and right knee flexion measures.
  - F_range is the target flexion range for Kiba Dachi.
  - D_kiba_min is the minimum lateral separation used to ensure a stable wide stance.
- *Squat repetition counting* A repetition is counted when the movement transitions from a top state to a bottom state and returns to the top state:
  $$\:Top\:\iff\:\:(F\_knee\:\ge\:\:F\_top)$$
  $$\:Bottom\:\iff\:\:(F\_knee\:\le\:\:F\_bottom)$$
  Repetition is counted when:
  $$\:Top\:->\:Bottom\:->\:Top$$
  where:
  - F_knee is the knee flexion measure computed from the hip-knee-ankle angle (use average of both knees or the minimum of both knees, but state which one you use).
  - F_top and F_bottom are the flexion thresholds defining the two states.
  - The “in sequence” requirement matches what you already state about counting repetitions through both threshold states.
- *Push-up repetition counting* A repetition is counted when the movement transitions from a top state to a bottom state and returns to the top state:
  $$\:Top\:\iff\:\:(F\_elbow\:\ge\:\:F\_top)\:AND\:(theta\_body\:in\:T\_plank)$$
  $$\:Bottom\:\iff\:\:(F\_elbow\:\le\:\:F\_bottom)\:AND\:(theta\_body\:in\:T\_plank)$$
  Repetition is counted when:
  $$\:Top\:->\:Bottom\:->\:Top$$
  where:
  - F_elbow is the elbow flexion measure computed from the shoulder-elbow-wrist angle. If you track both arms, define F_elbow as either:
    - The average of left and right elbow angles, or.
    - The minimum of the two (more conservative).
  - Pick one and state it explicitly.
  - F_top is the elbow-angle threshold defining the top position (arms extended).
  - F_bottom is the elbow-angle threshold defining the bottom position (arms flexed).
  - theta_body is the trunk alignment indicator (for example, shoulder-hip-ankle angle or trunk inclination).
  - T_plank is the allowed trunk-alignment tolerance window used to ensure the body remains approximately straight (plank-like posture).

These decision rules were designed to support feasibility testing and to enable transparent interpretation of the system output. However, their generalizability across body types and technique variations requires multi-participant validation.

### Training exercise detection

**Fig. 8:**
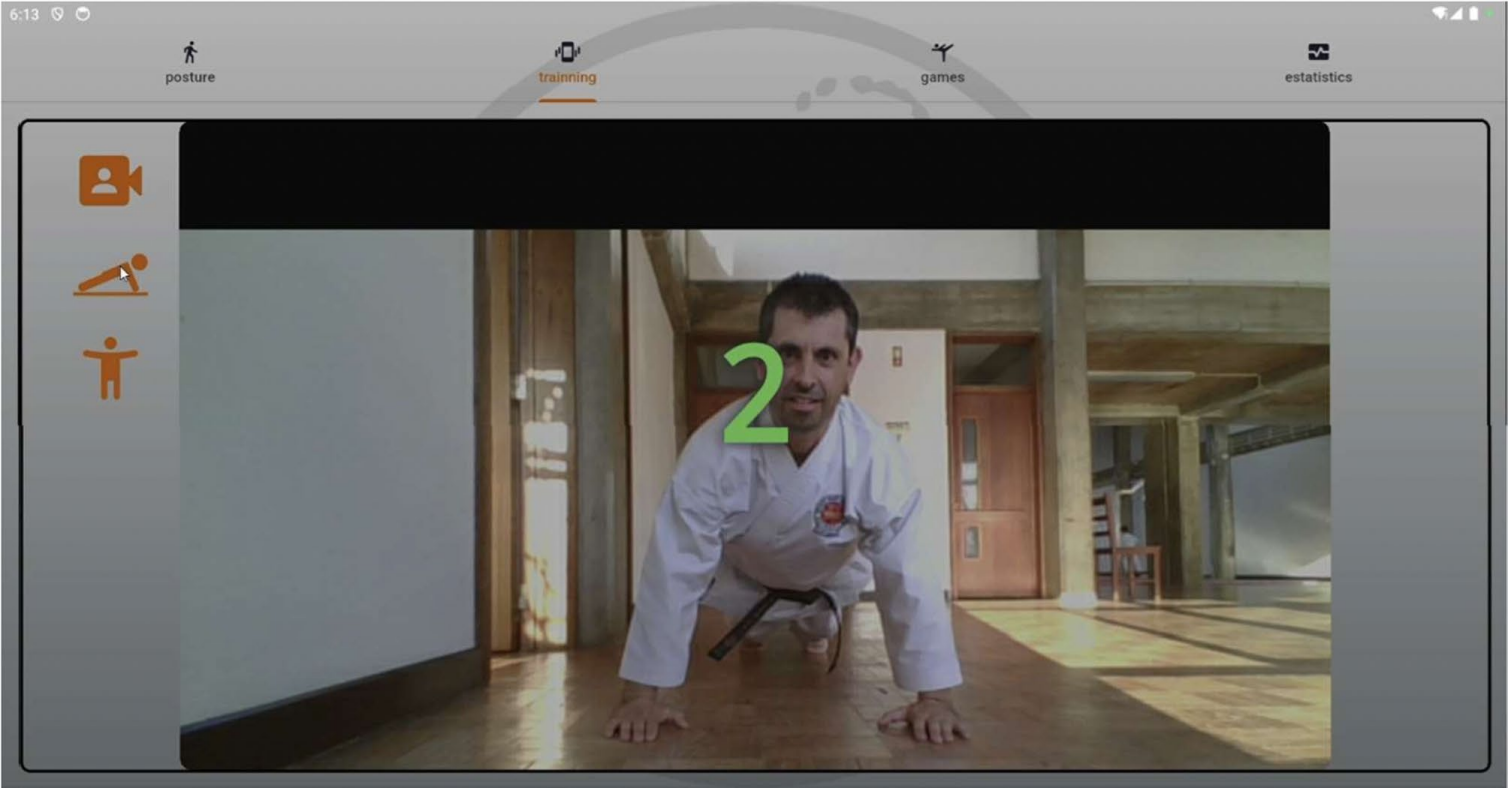
Push-ups detection.

**Fig. 9:**
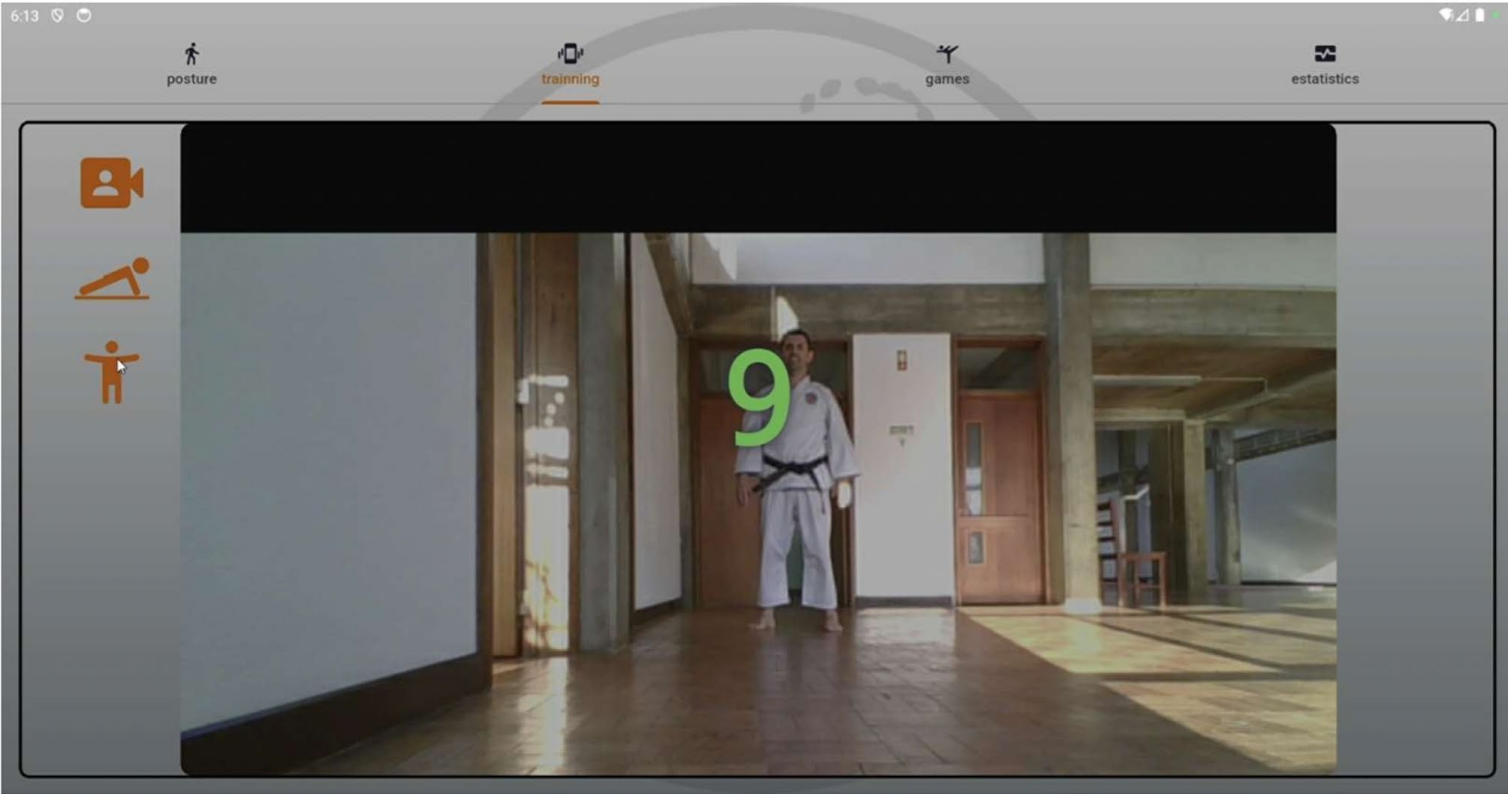
Squats detection.

To assist with conditioning, two exercises, push-ups and squats, were implemented using the same pose detection infrastructure. Repetitions are counted based on transitions between top and bottom positions:

- *Push-ups* Arm extension (elbow angles) distinguishes the upward and downward phases of the movement (Fig. 8).
- *Squats* Knee angles are tracked to determine squat depth (Fig. 9).

The app registers a successful repetition when the movement pattern passes through both threshold states in sequence.

### Gameplay mechanics

A basic reflex-testing game was implemented as an additional interactive module. In this game, users are prompted to perform a punch (tsuki) when a target appears on screen. The camera detects the strike from the forward motion of the hand, and the app calculates the reaction time.

Because of limitations in mobile camera frame rate (30 fps), this feature’s responsiveness was affected by latency, underscoring the need for optimisation on higher-performance devices.

### Data storage and feedback

**Fig. 10:**
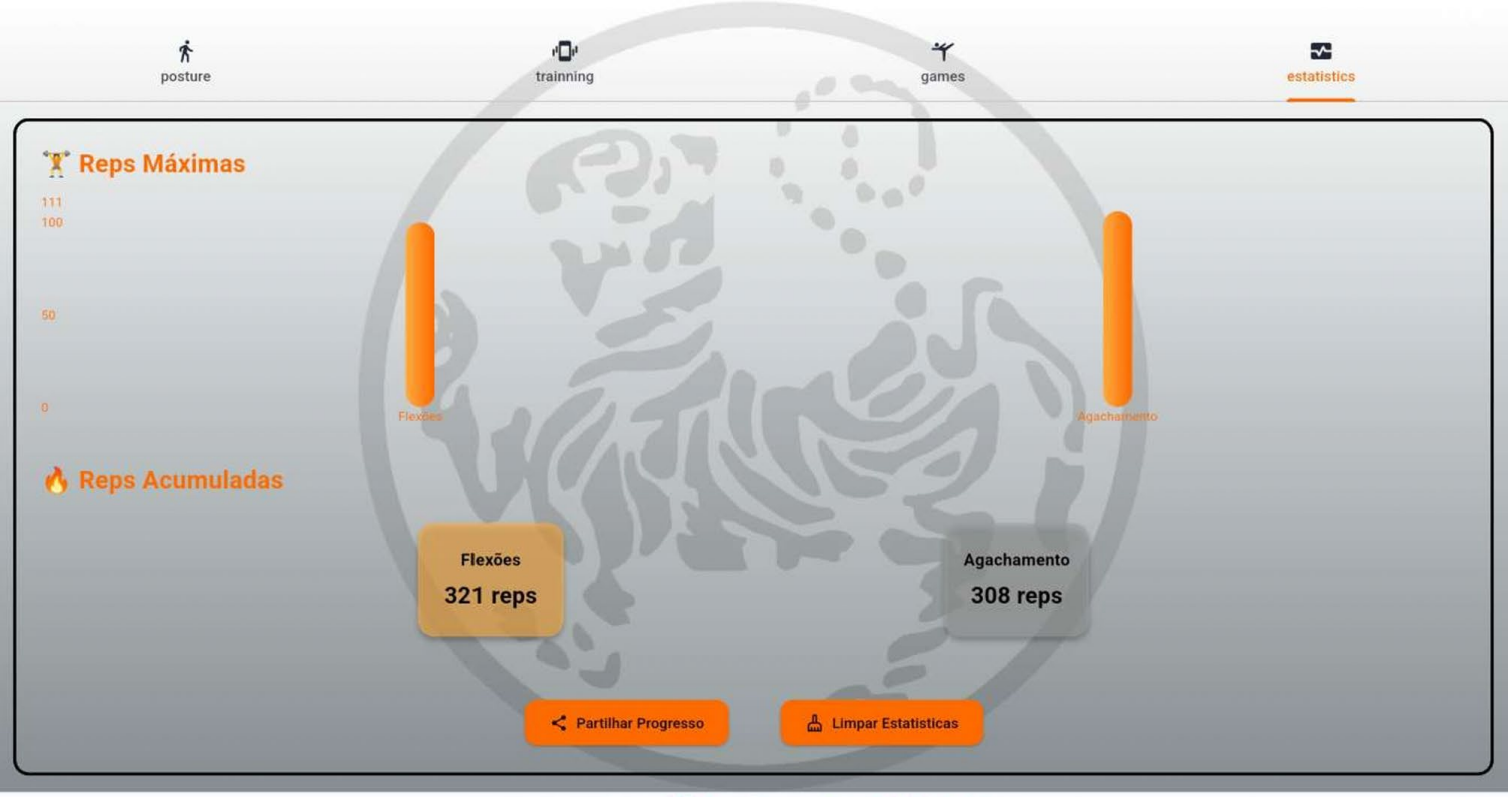
Statistics screen.

As shown in Fig. 10, after each session, the application:

- Stores repetition counts, posture validation results, and timestamps.
- Aggregates the data into a *statistics module* that tracks personal progress.
- Offers visual feedback through icons, scores, and charts.

Users can also share their progress via integrated social media sharing, enabling remote feedback and increased motivation.

### Ground truth and annotation protocol

To contextualise the quantitative comparisons reported in this feasibility pilot, we define the reference here as expert-provided labels and reference angle estimates obtained from video frames of the single participant. The purpose of this reference was to provide an initial feasibility check of whether app-derived stance labels and joint-angle features are broadly consistent with traditional Shotokan Karate posture criteria under controlled conditions, rather than to establish a validated biomechanical gold standard.

A single expert annotator was involved in the present pilot: a Shotokan Karate instructor with extensive teaching experience and familiarity with stance evaluation in practice. No additional annotators participated in the current study. The expert annotator was not blinded to the study objective. Annotations were not designed as an independent, blinded ground-truth procedure; therefore, they should be interpreted as an expert reference for feasibility assessment rather than as a fully objective measurement standard.

In this feasibility pilot, expert annotations serve as a pragmatic proxy reference (proxy ground truth) to support a preliminary comparison under controlled conditions, rather than as a definitive biomechanical gold standard. Definitive biomechanical validation would require instrumented reference systems (for example, optical motion capture and/or instrumented force measurement), which are outside the scope of the present feasibility pilot study and are planned for future work.

Frames were selected from recordings of the single participant performing Zenkutsu Dachi, Kokutsu Dachi, and Kiba Dachi under the controlled setup described earlier. The number of annotated frames per stance is reported in subsection 4.11. All frames originate from the same participant and should be treated as repeated measures within one subject, not as independent samples for inferential claims.

Angle comparisons in this study focused on lateral-view, sagittal-plane features derived from the pose landmarks. The knee angle is defined as the inter-joint angle at the knee formed by the hip, knee, and ankle landmarks, representing knee flexion in the sagittal plane, given the lateral camera setup. The shin angle is defined as the inclination of the shank segment relative to the vertical reference direction, computed from the knee and ankle landmarks. Because the setup uses a single 2D lateral view, frontal-plane and transverse-plane components such as abduction or rotation are not captured as true 3D biomechanical angles and are not treated as ground truth in this pilot.

The expert reference angles were obtained by frame-by-frame review of still frames, using a digital annotation workflow and visible anatomical landmarks as reference points. Since this pilot study did not include instrumented clinical measurement, no goniometer or physiologist-supervised simultaneous measurement was performed. The reported angle differences quantify disagreement between app-derived angles and the expert reference in this single-participant pilot study under controlled conditions.

Inter-rater and intra-rater reliability were not assessed in the present study because only one annotator contributed reference labels and angle estimates. Reliability assessment using multiple independent annotators and standardised repeated annotation will be included in the planned multi-participant validation protocol, together with metrics such as ICC for angles and agreement measures for stance labels.

All recordings in this pilot involved a single person in the camera field of view. Multi-person scenarios and person selection behaviour were not evaluated. Future work will explicitly test robustness in dojo-like conditions where additional people may enter the frame and assess the pose-tracking behaviour of the ML pipeline under multi-person conditions.

### Assumptions and constraints

The suggested system is a feasibility-oriented prototype, as it functions under explicit assumptions that directly limit the behaviour and applicability of the underlying algorithms. These presumptions are not coincidental; instead, they result from the combination of rule-based stance classification with a single-camera, 2D pose-estimation pipeline. Violations of these presumptions should be regarded as outside the purview of the current pilot study since they could result in reduced performance or failure modes.

The system is based on a single, laterally oriented camera roughly 90 degrees perpendicular to the practitioner’s sagittal plane. Because joint angles and segment orientations are calculated from 2D landmark coordinates projected onto the image plane, this assumption is necessary. Deviations from this perspective, such as camera rotation around the vertical axis or oblique views, cause perspective distortion that modifies apparent joint angles and renders the rule-based classifier’s fixed angular thresholds invalid. Consequently, dynamic camera motion and arbitrary camera viewpoints are not supported by the current implementation.

The algorithm assumes that the sagittal plane is where the main motion relevant to stance evaluation occurs. As a result, sagittal-plane flexion or inclination measurements obtained from a lateral projection are used to interpret joint angles. Abduction, adduction, and axial rotation are examples of frontal-plane and transverse-plane components that are not recorded as actual biomechanical quantities. This depth estimate is not metrically calibrated, despite the underlying pose-estimation model providing a relative Z-coordinate. As a result, the current system does not use it to calculate 3D joint angles. As a result, movements with significant out-of-plane motion may result in incorrect stance classification or angle estimates.

The system assumes there is sufficient, consistent lighting for the pose-estimation model to reliably identify body landmarks. In addition to causing jitter or the loss of important joints, poor lighting, strong shadows, motion blur, or visually complicated backgrounds can lower landmark confidence. Such circumstances could result in missed stance evaluations or false detections, as the rule-based layer relies on stable landmark positions to set thresholds. This study does not address robust operation in low-light or highly variable visual conditions.

The algorithm assumes that the hips, knees, and ankles, three important joints for stance evaluation, are visible in every analysed frame. Angle computation and threshold evaluation are directly impacted by unstable or absent landmarks caused by partial occlusion, self-occlusion, or occlusion by external objects. Beyond what the pose-estimation API provides, the current system lacks explicit occlusion handling and landmark confidence weighting.

Every recording used in this study features a single subject in the camera’s field of view. To retrieve landmarks for a single, most notable person, the system uses the pose-estimation API. Scenarios involving multiple people, like those frequently found in dojo settings, were not assessed. In these situations, stance evaluation may be jeopardised by improper person selection or identity switching, which is clearly outside the purview of the current implementation.

The empirically determined fixed angular and geometric thresholds used in the stance classification logic are fixed thresholds for this feasibility study. These thresholds do not specifically account for individual variations in body proportions, flexibility, or technique execution. As a result, without recalibration or adaptive thresholding, the existing guidelines might not apply to practitioners with various anthropometric traits. Future research will address anthropometric normalisation and personalisation.

In conclusion, the operational envelope within which the suggested system operates as intended is defined by the aforementioned assumptions. Additionally, they motivate future extensions such as adaptive thresholding, multi-view or depth-based analysis, multi-person handling, and robustness testing under various environmental conditions, and they directly explain the algorithmic limitations found in the pilot study.

### Quantitative evaluation

The quantitative evaluation compared the app-derived joint-angle estimates (knee and shin) and stance labels against expert reference annotations derived from 2D video frames of a single participant, as an initial feasibility check rather than a biomechanical gold-standard validation. This comparison was performed using a set of labelled images and videos covering three key Shotokan Karate stances: Zenkutsu Dachi (front stance), Kokutsu Dachi (back stance), and Kiba Dachi (horse stance).

For each stance, the number of samples (images/frames) used for the calculation was as follows:

- Zenkutsu Dachi: 100 frames.
- Kokutsu Dachi: 95 frames.
- Kiba Dachi: 90 frames.

The app generated stance labels and joint-angle estimates in this one-person trial, which were then compared with expert reference annotations. This pilot study’s results are preliminary and not generalisable. The results cannot be generalised due to the small sample size. These findings should be regarded as a feasibility pilot study until a larger sample is tested. Additionally, validation with more participants is necessary.

The app’s output was compared against expert reference annotations provided by a Shotokan Karate instructor (expert annotator) to assess consistency with Shotokan stance criteria in this feasibility pilot. In the planned multi-participant validation, we will additionally collect objective reference angle measurements using digital goniometry (manual goniometer and/or image-based angle measurement software) and, where available, an RGB-D camera (for example, Kinect-class devices) to benchmark app estimates. To determine whether the app’s feedback aligned with conventional Shotokan Karate technique criteria, postures and joint angles were compared against expert reference annotations (proxy ground truth) derived from 2D video frames in this single-participant feasibility pilot. To evaluate consistency with conventional Shotokan Karate posture criteria, the stance detection and joint angle estimates were compared against these expert annotations. Nevertheless, only one participant participated in this validation, so the findings should be regarded as preliminary. To ensure broader applicability, future research will increase the sample size with a minimum of 30 Shotokan Karate practitioners across three skill strata (beginner, intermediate, advanced) and incorporate statistical error measures, such as RMSE and Bland-Altman analysis, to more accurately evaluate the app’s measurement agreement and accuracy.

To improve the robustness of the study, we propose conducting future testing with a larger sample size, which will allow for the inclusion of error and agreement metrics, such as:

- Root Mean Square Error (RMSE) to assess the accuracy of the app’s measurements.
- Confidence Intervals (CI) to provide a range for potential errors and assess the variability of the app’s output.
- Correlation Coefficients to measure the relationship between the app’s results and expert-provided ground truth.
- Bland-Altman Analysis to assess the level of agreement between the app’s measurements and the expert’s assessments.

These metrics will provide a more complete picture of the app’s performance and improve the reliability of the conclusions.

### Computational performance and latency profiling

We included an engineering-focused performance profile of the mobile pipeline to support the reproducibility of the real-time claim. The time interval between the camera frame timestamp at acquisition and the moment posture or game feedback is displayed on the screen is what we refer to as end-to-end latency. Additionally, we report per-frame processing time as the total of the rule-based post-processing time (angle calculation and stance/exercise decision logic) and the pose-estimation inference time. Furthermore, we report the percentage of dropped or skipped frames for each test condition, along with the observed effects on the processed frame rate.

A specific set of mobile devices, representative of mid-range and higher-performance smartphones, was used for profiling. We used system monotonic clocks to record timing logs while running the posture and reflex-game modules on each device for a predetermined period in controlled indoor environments. The median end-to-end latency (P90), median per-frame processing time, and achieved processed frames per second are reported for each condition. We specifically list device models, OS versions, camera resolution, and target fps settings used during profiling because camera configuration and OS scheduling can impact timing.

These findings describe the prototype’s responsiveness and shed light on the 30 fps capture’s present limitations for quick movements. Future research will expand profiling to more variable real-world conditions and higher frame-rate capture modes (e.g., 120 to 240 fps when supported).

## Results

This section reports the outputs produced by the prototype under the controlled conditions of the single-participant pilot. The results cover three core functional areas: posture analysis, training exercise validation, and interactive gameplay. Outputs were generated under the controlled conditions described in Section “Methodology”, and quantitative comparisons were computed from a predefined set of extracted frames as described in Subsection “Evaluation protocol and reproducibility”.

### Posture analysis performance

The posture module generated pose landmarks and derived joint-angle features (hip, knee, ankle angles, and segment inclinations) from the lateral camera view under the tested indoor conditions. These features were used by the rule set to assign stance labels for the three target stances in this pilot. Using ML Kit Pose Detection, landmarks were extracted under standard lighting and static background conditions. The analysis of joint angles and body alignment features was used by the rule set to assign stance labels to stance configurations, as implemented in this pilot.

The system (i) identified the front and back leg using foot position, (ii) computed hip, knee, and ankle angles from pose landmarks, and (iii) computed a trunk-bias proxy from the trunk axis intersection with the base of support. However, several limitations emerged. The initial algorithms relied on assumptions, such as the practitioner having a centred torso, which led to false negatives in asymmetric poses. Subjective posture cues, such as the perceived feel of a stance, could not always be translated into algorithmic thresholds. In addition, variability in body proportions across users made it difficult to define generalisable fixed-angle tolerances. These observations led to iterative refinement of the algorithms, prioritising relative measurements (e.g., ratios, body segment alignment) over fixed absolute values.

The results presented in this study are based on pilot testing with a single participant. The app produced outputs consistent with the implemented rules under the tested conditions in posture analysis and exercise tracking, but further testing with a larger group is necessary to assess the generalizability of these results.

### Training exercise evaluation

The application detected and counted repetitions of basic training exercises under the tested conditions:

- Push-ups were evaluated based on the elbow angle variation between the top and bottom positions.
- Squats were tracked using knee angle thresholds to detect full depth and return.

The evaluation results showed consistent detection of exercise phases when movements were performed within a clear range, repetition counts matched the threshold-state logic when joints remained visible in the camera frame, and smooth visual feedback with immediate logging of results in the statistics module. However, several limitations were observed. Exercise detection performance in this pilot was assessed qualitatively rather than through formally defined rep-level error rates. Although false positives and false negatives were observed during development and testing, a fixed experimental protocol defining the repetition-level unit, reference standard, and denominator was not established for this feasibility pilot study evaluation. As a result, quantitative rep-level error rates (e.g., false-negative percentages) are not reported in this study.

Qualitative failure modes included missed repetitions when key joints (such as elbows or knees) were partially occluded or moved out of the camera frame, and spurious detections when users paused mid-exercise and momentarily satisfied threshold conditions. These observations motivate future improvements but should not be interpreted as validated error rates.

In future multi-participant validation, exercise performance will be evaluated at the repetition level using a formally defined protocol. The experimental unit will be one attempted repetition, with reference repetition counts obtained through manual video review. False positives and false negatives will be defined at the rep level using a temporal tolerance window. To reduce transient false positives, a time-windowed state machine with hysteresis and a refractory period will be implemented and evaluated using an ablation design (pre- vs. post-temporal smoothing), with rep-level precision and recall reported across varied conditions.

### Game performance

The reflex-based punch-detection game allowed users to perform a timed strike toward an on-screen target. However, technical constraints affected responsiveness. Thus, punch detection was based on tracking forward hand movement relative to the torso, and response time was calculated from the target display event to the detection of the strike.

In practice, the application detected forward hand motion events corresponding to punch attempts under the tested conditions. Still, latency was a major issue, mainly due to the 30 frames-per-second camera rate and mobile processing delays. On lower-end devices, this lag was sufficient to affect the measured reaction-time outputs and user experience of the reflex test.

### Usability and interface feedback

The application adopted a minimalist design with straightforward navigation and iconography, prioritising function over aesthetic complexity. Informal feedback collected during development indicated that users found the interface clear and the visual helpful feedback. However, no structured usability study was conducted, and these observations should be interpreted as anecdotal rather than as a systematic evaluation.

No dedicated accessibility features were implemented or evaluated in this prototype (e.g., screen reader support, scalable text, colour contrast checks, haptic cues, or alternatives to colour-only feedback). These are priorities for future iterations. Planned improvements include support for system font scaling, high-contrast mode, haptic confirmation for feedback events, captions and non-audio cues, and ensuring compatibility with platform screen readers. A structured usability evaluation will accompany these updates.

### Quantitative performance analysis

The app’s stance labels and joint-angle estimates were evaluated by comparing its output against expert reference annotations obtained from 2D video frames in this single-participant feasibility pilot. These comparisons are descriptive and not a biomechanical gold-standard validation. The results include the app’s detected knee and shin angles, as well as the stance classification for each image. The following data provides insights into the app’s performance across various stances and joint angles. Table 1 summarises representative frame-level comparisons extracted from static posture holds in the single-participant pilot and is provided for illustrative purposes rather than for statistical inference. All frames were extracted from recordings of a single participant and should not be interpreted as independent samples.

**Table 1:** Comparison of app-detected and expert-annotated joint angles and stance classifications.

Side detected	Correct side?	Stance detected	Expert annotation (Stance)	Left leg knee angle (°)	Left leg shin angle (°)	Right leg knee angle (°)	Right leg shin angle (°)
Right	Yes	Zenkutsu Dachi	Zenkutsu Dachi	9	144	65	63
Left	Yes	Zenkutsu Dachi	Zenkutsu Dachi	33	91	11	136
Left	No	Kiba Dachi	Kiba Dachi	34	105	25	105
Left	Yes	Kokutsu Dachi	Kokutsu Dachi	16	109	13	102
Right	Yes	Zenkutsu Dachi	Zenkutsu Dachi	1	161	70	121
Left	Yes	Zenkutsu Dachi	Zenkutsu Dachi	38	94	29	110
Left	Yes	Kokutsu Dachi	Kokutsu Dachi	33	97	13	130
Left	Yes	Kiba Dachi	Kiba Dachi	45	120	26	107
Right	Yes	Kokutsu Dachi	Kokutsu Dachi	7	118	18	115

### Error analysis

We compared the app-estimated knee and shin angles with expert reference annotations to characterise the magnitude of disagreement observed in this single-participant pilot. Error statistics were computed over *N* = 9 analysed frames/images, where each sample corresponds to one video frame with expert-annotated angles. Frames were selected using uniform sampling across each stance hold or the frames listed in Table 1. Because all samples originate from a single participant, these values are descriptive only and should not be interpreted as generalisable performance.

We report mean absolute error (MAE) in degrees (deg), computed as depicted in Eq. (1):

1 $$\:MAE=\frac{\sum\:\left|\left.{{\uptheta\:}}_{\mathrm{a}\mathrm{p}\mathrm{p}}-{{\uptheta\:}}_{\mathrm{e}\mathrm{x}\mathrm{p}\mathrm{e}\mathrm{r}\mathrm{t}}\right|\right.}{N}$$

where θ_app_ is the app-estimated angle, and θ_expert_ is the expert-annotated angle for the same frame. No outlier exclusion was applied; all selected frames were included in the analysis.

Using this definition, the following descriptive values were obtained:

- Knee angle MAE: 5.3° ± 3.2°.
- Shin angle MAE: 4.8° ± 2.6°.

These values should be interpreted as illustrative only and do not represent validated accuracy or generalisable performance. Disagreement may increase under partial occlusion, camera placement deviations, lighting variation, or differences in body proportions, which will be assessed in future multi-participant validation. In future work with a larger dataset, we will also report distribution summaries (e.g., median/P50 and P90 absolute errors) and agreement analyses (e.g., Bland–Altman plots), together with subject-wise reporting and confidence intervals.

### Summary of results

Table 2 summarises the evaluation of the prototype application in four areas: Posture Analysis, Exercise Detection, Gameplay, and Usability. The system generated frame-level joint-angle estimates under controlled conditions, with the descriptive error values reported above. Empirically tuned thresholds were applied to assign stance labels in this pilot dataset. However, it faced challenges like subjective assessments, user variability, and torso alignment issues. The detection was visibility-dependent, and frame-rate latency increased timing uncertainty. Usability findings were preliminary due to limited testing and a lack of accessibility features, indicating priorities for future iterations.

**Table 2:** Application results.

Feature area	Strengths	Limitations
Posture analysis	Frame-by-frame joint-angle estimates under controlled conditions	Subjectivity, user variability, and torso alignment issues
Exercise detection	Repetition events detected using threshold-state transitions	Visibility-dependent; false positives observed during pauses
Gameplay	Target-triggered punch event detection and reaction-time computation	30 fps capture and processing latency increased timing uncertainty
Usability	Simple UI, easy access to features	Limited testing, no accessibility features included

### Evaluation limitations

The primary limitation of this evaluation is the small sample size, as the entire quantitative evaluation is based on a single participant. This limits the ability to make any meaningful statistical conclusions about the accuracy, reliability, or validity of the mobile application. Because the evaluation involved a single participant and controlled capture conditions, external validity is not established, and the results cannot be generalised across practitioners, body types, or recording environments. Given that the pilot testing was conducted with only one participant, the results should be regarded as preliminary and illustrative, rather than definitive. Also, only a single expert instructor was involved in the present pilot, and no inter- or intra-rater reliability analyses were performed.

To address these limitations, future studies should involve larger sample sizes to enable more meaningful statistical analyses and increase the reliability of the findings. A statistical justification for the sample size should be provided using an a priori power analysis (e.g., G*Power) or by citing relevant literature. Future studies should also recalculate all quantitative metrics using the expanded dataset and incorporate reliability analyses, such as inter-rater, intra-rater, and between-method comparisons, to strengthen the study’s scientific foundation.

Until these data are collected and analysed, the results presented in this study should be clearly described as preliminary. These should not be used to support broader claims regarding the app’s accuracy or practical utility.

In future multi-participant studies, stance thresholds will be derived empirically rather than tuned manually by comparing expert-annotated stance labels and joint angles with app-generated estimates across a larger dataset. In the future, at least two independent expert annotators will label the same frames, enabling inter-rater and intra-rater reliability assessment (for example, ICC). Thresholds and decision rules will then be selected using a data-driven procedure (e.g., optimising stance classification agreement and reporting associated uncertainty), providing a more objective, statistically grounded basis for the classification cutoffs.

## Discussion

The creation of this mobile application demonstrates the expanding possibilities of incorporating mobile and computer vision technologies into conventional martial arts training. This project helps modernise how practitioners can train and improve their methods, particularly when access to a dojo or instructor is restricted, by converting subjective, instructor-led evaluations into algorithmic feedback mechanisms.

This pilot study demonstrates the feasibility of using smartphone-based pose detection to support self-guided Shotokan Karate training under controlled conditions. However, results must be interpreted cautiously because the quantitative evaluation involved only one participant (*n* = 1) and should not be extrapolated.

To validate the app’s performance across various skill levels, body types, and training objectives, future research should increase the sample size to include a more diverse group of participants. This will ensure more reliable findings on the app’s efficiency, precision, and usability.

### Contributions and strengths

One of the most notable achievements of the project is its successful transformation of traditional Shotokan Karate postures into computable parameters. The system’s ability to detect joint positions, calculate angles, and infer body alignment using only a smartphone camera suggests that this type of solution is viable on commodity smartphones and may be practical for real-world use. However, further multi-user testing is needed to confirm this.

The application also contributes in several ways: it enhances practitioner autonomy by allowing users to train with guidance beyond the dojo, promotes more objective feedback and reduces dependence on subjective human judgment, supports physical conditioning through integrated repetition tracking for basic exercises, and increases engagement by incorporating gamified elements and progress statistics.

By combining sport, education, and technology, the project offers an inclusive, personalised, and modernised approach to martial arts training.

### Technical challenges and limitations

While the system architecture and algorithms were functional in controlled scenarios, several limitations were identified. Posture detection accuracy was sensitive to user positioning, lighting, and partial occlusion; when key joints such as the knees or ankles were not clearly visible, performance decreased. In addition, the system relies on a single laterally positioned 2D camera view, so joint angles are derived from 2D projections rather than true 3D kinematics. As a result, out-of-plane motion, foot rotation, and variations in hip or trunk orientation can introduce projection error and reduce the reliability of joint angle estimates, particularly during stances or transitions that involve transverse-plane rotation or frontal-plane deviations. The subjective nature of karate technique also made it difficult to define universal threshold values, since posture correctness can vary across federations, styles, and individual body types. Algorithm rigidity was another concern, as the system relied on fixed tolerance margins for angles and positioning that may not accommodate practitioner diversity without configurable flexibility.

Regarding temporal resolution, the pilot implementation operated at 30 fps, which contributed to latency in the reflex-based game module and also limited the accuracy of pose estimation and joint angle extraction during rapid movements. Higher frame-rate capture, available on many modern smartphones (e.g., 120–240 fps), is expected to improve temporal precision and tracking stability for fast techniques. Additionally, due to the small user base and the lack of large-scale testing, empirical validation of the results was limited. Feedback from an expert instructor and future multi-participant studies with standardised annotation protocols and reliability assessment will be essential to refine the detection logic and verify alignment between algorithmic suggestions and human evaluation.

### Human factors and practicality

Karate is not only a physical discipline but also a cultural and pedagogical tradition. While this project introduces valuable technical support, it does not aim to replace the nuanced guidance of an experienced sensei. Rather, the tool functions as a complementary aid, handy for:

- Beginners who need visual references and validation of stance fundamentals;
- Practitioners training outside the dojo;
- Instructors seeking a supplementary teaching aid for individual feedback.

It is essential to recognise that training motivation, technique feel, and rhythm are challenging to quantify algorithmically. These aspects still require human interpretation and mentorship.

### Broader implications

This project aligns with a broader trend in digital transformation within sports education, particularly in areas such as remote coaching, performance analytics, and gamified learning, and its methodology and results could be extended to other martial arts such as Taekwondo, Kung Fu, and Judo, to rehabilitation and physiotherapy settings, and to home fitness applications that require motion feedback. Additionally, the application lays the groundwork for future integration with wearable sensors, AR overlays, or even AI-powered virtual instructors.

Beyond individual training, the proposed system also has relevance for remote and blended education in motor and sport sciences, where practice-oriented activities are difficult to supervise outside laboratory or dojo settings. Prior work has highlighted both the feasibility and the limitations of online sport sciences education, particularly the lack of structured feedback during unsupervised practical training. In this context, mobile vision-based posture analysis tools may serve as complementary educational technologies, providing consistent, instructor-informed feedback during autonomous practice. As noted by Iuliano et al.^32^, effective remote sport sciences education requires tools that help bridge the gap between theoretical instruction and practical execution. Although the present study is limited to a single participant, it illustrates how accessible, camera-based systems could support blended learning models by extending structured movement feedback beyond face-to-face supervision.

### Implications of quantitative results

When interpreting the quantitative results, it is important to reiterate that the reported angle-disagreement values and stance-classification behaviour reflect a single-participant feasibility pilot conducted under tightly controlled conditions. Specifically, recordings were obtained with a fixed lateral camera viewpoint, controlled indoor lighting, and primarily quasi-static stance holds. Therefore, the reported error margins should be interpreted as valid only within this constrained setup and should not be generalised to other participants, camera viewpoints, lighting conditions, dynamic transitions, or real dojo environments. A multi-participant validation with varied capture conditions and independent annotation will be required to establish generalisable performance.

The quantitative results provide preliminary, illustrative evidence that the app can identify key Shotokan Karate stances and estimate joint angles under controlled conditions in a single-participant pilot. The mean errors of 5.3º for knee angles and 4.8° for shin angles illustrate the magnitude of disagreement observed in this single-participant pilot example. In this pilot example, the rule-based classifier correctly matched expert stance labels in most analysed frames, indicating the approach’s feasibility. The observed knee angle disagreement of approximately ± 5.3° illustrates the magnitude of differences observed in this single-participant pilot. Despite these encouraging results, some limitations were noted. The app’s performance can be influenced by factors such as lighting, camera angle, and partial body occlusion. For instance, when certain parts, such as knees or elbows, are outside the camera frame, the app may miss repetitions or record incorrect angles. Future improvements could include optimising the system for different body types, improving accuracy when parts are partially hidden, and resolving frame rate issues in the reflex-based game module.

### Lessons learned

Several lessons emerged during development. First, simplicity and usability are critical, as users need clear visual cues and immediate, interpretable feedback. Second, the shift from instructor intuition to algorithmic evaluation requires careful translation of technique into geometric and mathematical models. Finally, adaptability should be built into posture evaluation systems so they can be configured for different styles and individual users.

## Conclusion

This project aimed to develop a mobile application that integrates pose detection, on-device feedback, and interactive training to support Shotokan Karate practitioners in improving both their technique and physical conditioning. By utilising Google’s ML Kit for pose estimation and translating traditional martial arts principles into algorithmic logic, the application offers an innovative tool for evaluating key postures, such as Zenkutsu Dachi, Kokutsu Dachi, and Kiba Dachi.

Given the limitations of this single-participant pilot study, the results demonstrate the feasibility of assessing fundamental stances under controlled conditions using mobile devices and on-device processing, while generalisable reliability requires multi-participant validation. These findings illustrate the feasibility of stance classification in a controlled single-participant pilot, but they do not establish validated accuracy or generalisable performance. The application also produced outputs consistent with the implemented rules under the tested conditions for detecting and counting repetitions for basic conditioning exercises. Additionally, it provided an engaging, though latency-affected, reflex-based game that enhanced both physical and mental training.

While the application represents a meaningful step toward modernising martial arts education, it also highlights several opportunities for future development:

- *Improving accuracy and flexibility*: The current system uses fixed angular thresholds that may not adequately accommodate variations in body type or stylistic differences. Future iterations should include configurable tolerance levels and adaptive learning based on user feedback and expert annotations.
- *Expanding movement analysis*: At present, the system focuses exclusively on static stances. A logical next step would be to analyse dynamic techniques, such as punches (tsuki) and kicks (geri), and transitional movements in kata, using temporal analysis and sequence modeling.
- *Enhanced game mechanics*: The reflex game component, while promising in concept, requires optimisation. Improving frame rate handling or integrating motion sensors (e.g., accelerometers or wearables) could enhance response accuracy and expand game modes.
- *Larger-scale testing*: Wider deployment and systematic testing in real-world dojo environments with diverse practitioner populations would yield valuable data on performance, usability, and acceptance. Integration with instructor feedback would also validate the tool’s educational value.
- *Cross-disciplinary integration*: Beyond karate, the approach has broader potential for application in other martial arts, physical therapy, or general fitness, particularly in remote or underserved contexts where access to professional instruction is limited.

In conclusion, this work suggests that accessible, mobile-based technologies can complement traditional martial arts training by providing immediate, objective feedback through a familiar, user-friendly interface. While not a replacement for human instruction, the system offers a powerful supplemental tool for students and instructors alike, supporting the evolution of karate teaching into the digital age.

## Data Availability

De-identified derived data supporting the pilot analyses (for example, annotated joint angles, per-frame error calculations, and summary tables) and the evaluation script will be made available on reasonable request. Raw video and identifiable images are not publicly shared due to privacy constraints, but may be reviewed under a controlled-access agreement, subject to consent and institutional policy.
